# Role of mitochondrial reactive oxygen species in homeostasis regulation

**DOI:** 10.1080/13510002.2022.2046423

**Published:** 2022-02-25

**Authors:** Baoyi Zhang, Cunyao Pan, Chong Feng, Changqing Yan, Yijing Yu, Zhaoli Chen, Changjiang Guo, Xinxing Wang

**Affiliations:** a Tianjin Institute of Environmental and Operational Medicine, Tianjin, People’s Republic of China; b Department of Public Health, Lanzhou University, Lanzhou, People’s Republic of China; c School and Hospital of Stomatology, Tianjin Medical University, Tianjin, People’s Republic of China

**Keywords:** Mitochondrial reactive oxygen species, signal transduction, aging, stem cells, hypoxia, cell differentiation, oxidative stress, electron transport chain

## Abstract

Mitochondria are the main source of reactive oxygen species (ROS) in cells. Early studies have shown that mitochondrial reactive oxygen species (mROS) are related to the occurrence and adverse outcomes of many diseases, and are thus regarded as an important risk factor that threaten human health. Recently, increasing evidence has shown that mROS are very important for an organism’s homeostasis. mROS can regulate a variety of signaling pathways and activate the adaptation and protection behaviors of an organism under stress. In addition, mROS also regulate important physiological processes, such as cell proliferation, differentiation, aging, and apoptosis. Herein, we review the mechanisms of production, transformation, and clearance of mROS and their biological roles in different physiological processes.

## Introduction

**Figure 1.**
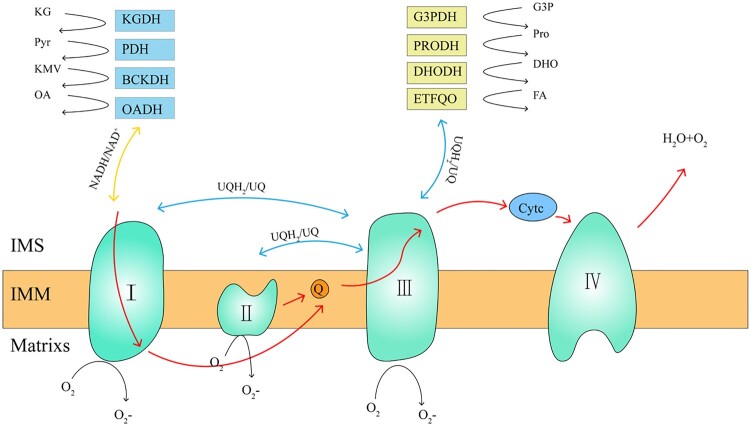
**mROS production sites and mitochondrial electron transfer process.** The mROS generation sites can be divided into two categories, namely NADH/NAD^+^ equipotential group (yellow) and the UQH_2_/UQ equipotential group (blue). The NADH/NAD^+^ group consists of KGDH, PDH, BCKDH, OADH, and complex I. The UQH_2_/UQ isopotential group is made up of complex II, PRODH, DHODH, ETFQO, and complex III. Complex I uses two equipotential groups to form reactive oxygen species. The red line indicates the electron transfer process of the mitochondria. mROS, mitochondrial reactive oxygen species; UQH_2_, ubisemiquinone; UQ, ubiquinone; KGDH, α-ketoglutarate dehydrogenase; PDH, pyruvate dehydrogenase; BCKDH, branched chain keto acid dehydrogenase; OADH, 2-oxoadipate dehydrogenase; PRODH, proline dehydrogenase; DHODH, dihydroorotate dehydrogenase; and ETFQO, electron transferring flavoprotein ubiquinone oxidoreductase.

Reactive oxygen species (ROS) are by-products of cell aerobic respiration. There are many kinds of ROS, including superoxide anion (O_2_^-^), hydrogen peroxide (H_2_O_2_), hydroxyl radical (HO·) and nitric oxide (NO), et al. NO also belongs to the RNS category. At present, the researches mainly focus on H_2_O_2_ and O_2_^-^. However, HO· produced by H_2_O_2_ and Fe^2+^ through the Fenton reaction is also an important type of ROS. ROS are generally regarded as toxic metabolites [1], and one of the driving factors of cancer [2–5], diabetes [6–8], and cardiovascular diseases [9,10]. However, ROS has been proposed as an active factor regulating many kinds of life activities since the 1990s. For example, cytokines, insulin, growth factors, AP-1, and NF-KB signals all require H_2_O_2_ [11]. Subsequent studies have shown that ROS play an important role in a wide range of physiological processes such as cell proliferation and differentiation, gene expression, post-translational protein modification, homeostasis and hypoxia adaptation [12–18]. Therefore, in the normal physiological state, cells maintain a certain level of ROS to ensure homeostasis [19] Figure 1.

Mitochondria are the main location of aerobic respiration to supply energy and are the main source of ROS in cells [20–22]. The initial conception that mitochondrial ROS are essentially undesirable metabolites generated by the cellular respiratory chain has changed. A large body of experimental evidence indicates that mitochondrial reactive oxygen species (mROS) production is a continuous and tightly regulated process required for the regulation of many life activities [23]. mROS have been proven to be involved in the regulation of many physiological processes, such as cell differentiation, senescence, signal transduction, hypoxic adaptation [13–18,24–26]. For example, Liu et al. [27] showed that mROS can regulate the release of cytochrome c and thus mediate apoptosis. Chandel et al. [24] revealed that in the hypoxic environment, mROS can promote the production of hypoxia inducible factors (HIFs) by regulating the expression of multiple functional genes, to initiate a protective mechanism of cells against hypoxia, thus playing an important role in the regulation of hypoxia homeostasis. Subsequently, it was found that H_2_O_2_ released by mitochondria can activate signal factors such as c-Jun amino-terminal kinase 1 (JNK1), p53, and nuclear factor kappa B (NF- κB) [25,26].

## The production of mROS

In the 1970s, researchers first found that isolated mitochondria could produce superoxide and H_2_O_2_ [28–32]. Since then, the production of mROS and its role in diseases have been studied in detail. The generation of mROS is mainly caused by the leakage of electrons in the electron transport chain (ETC) [33,34]. Under physiological conditions, 0.2–2% of the electrons in the ETC cannot be transferred normally, but leak out from ETC and interact with oxygen to form superoxide or H_2_O_2_ [28–32]. The ETC is composed of transmembrane protein complex (I–IV) in the mitochondrial crest, the free-moving electron transfer vector ubiquinone (UQ) and cytochrome c [34]. Together with F1F0 ATP synthetase (complex V), they become the basis of ATP production in oxidative phosphorylation (OXPHOS) [35,36].

Up to now, 11 mROS production sites have been found in mammalian mitochondria, which are related to substrate catabolism, electron transport, and OXPHOS. Six sites work at the redox potential of the NADH/NAD^+^ isopotential pool; and five sites work at the redox potential of the UQH_2_/UQ isopotential pool (Figure 1) [16,37]. The former is composed of a flavin-dependent dehydrogenase that reduces or oxidizes nicotinamide, mainly including α-ketoglutarate dehydrogenase (KGDH), pyruvate dehydrogenase (PDH), branched chain keto acid dehydrogenase (BCKDH), and 2-oxoadipate dehydrogenase (OADH), as well as the flavin mononucleotide group of complex I. The latter is composed of enzymes that directly oxidize or reduce mitochondrial UQ to produce ROS, including complex I, complex II, complex III, sn-glyceral-3-phosphate dehydrogenase (G3PDH), proline dehydrogenase (PRODH), dihydroorotate dehydrogenase (DHODH), and electron transferring flavoprotein ubiquinone oxidoreductase (ETFQO) [38].

## Regulation of mROS

**Figure 2.**
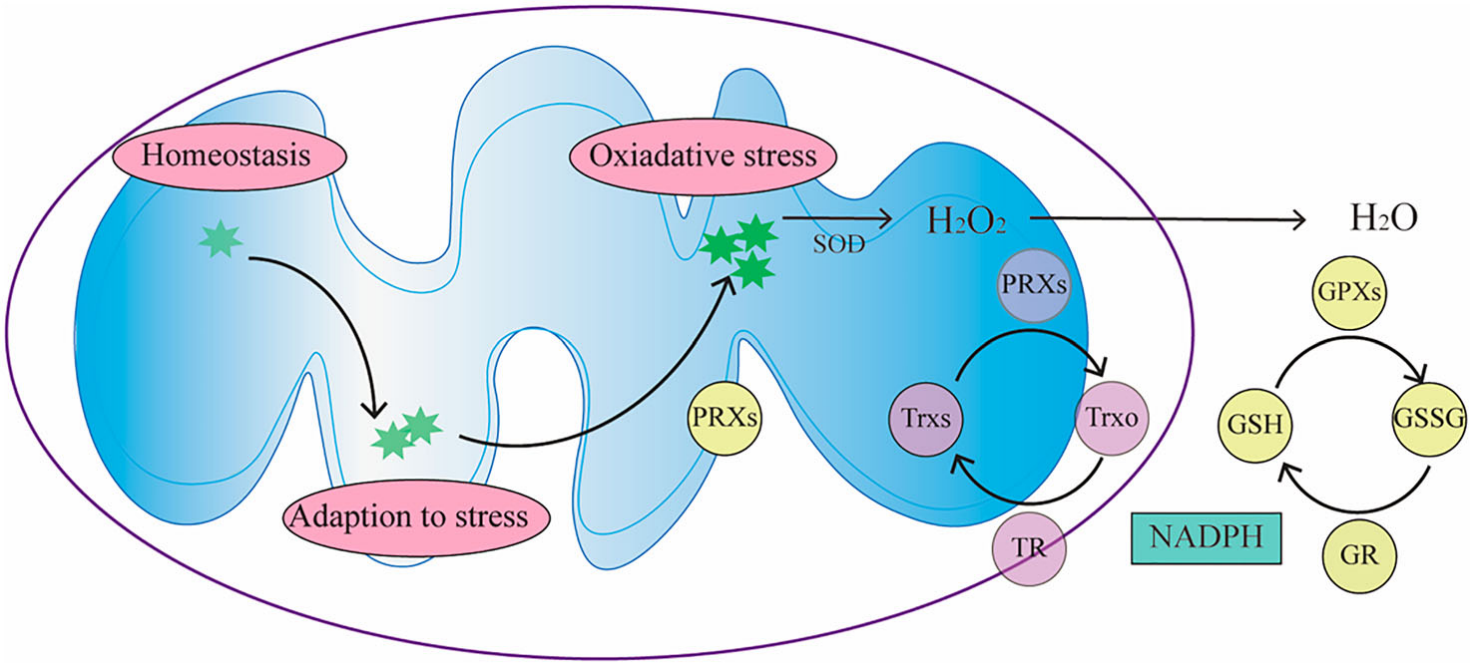
**Regulation of mROS.** The level of mROS determines mitochondrial function and physiological outcomes. The organism needs low levels of mROS to maintain homeostasis, and when mROS rise to higher levels, cells can adapt to stress in a variety of ways. When the mROS level accumulates to a very high level, oxidative stress and damage occurs. SOD can convert O_2_^-^ into H_2_O_2_. The generated H_2_O_2_ can be further converted into H_2_O by PRXs and GPXs. mROS, mitochondrial reactive oxygen species; SOD, superoxide dismutase; PRX, peroxiredoxin; GPX, glutathione peroxidase; TRXr/o, reduced/oxidized thioredoxin; GSH/GSSG, reduced/oxidized glutathione; TR, thioredoxin reductase; GR, glutathione reductase.

The *in vivo* level of mROS is related closely to their characteristics and physiological functions (Figure 2); therefore, there is a precise control system for mROS to maintain the balance between its production and elimination. mROS scavenging system can reduce oxidative stress damage [39]. This system mainly includes enzymatic and non-enzymatic parts. The non-enzymatic part of the system mainly consists of hydrophilic and lipophilic antioxidants, such as tocopherol, ascorbic acid, reducing coenzyme Q10 and glutathione. The antioxidant enzymes in cells mainly include superoxide dismutase (SOD), glutathione peroxidases (GPXs), peroxiredoxins (PRXs) and catalase (CAT). ROS, including mROS, can be efficiently eliminated, collectively referred to as the protective enzyme system. First, when the intracellular ROS concentration is high, the antioxidant defense system equipped by mitochondria can reduce the cytotoxicity caused by ROS. For example, O_2_^-^ can be efficiently dismutated to H_2_O_2_ by Mn^2+^-dependent superoxide dismutase (Mn-SOD) [40]. Then H_2_O_2_ is removed by PRXs and GPXs [28–32,41], which can be regenerated by glutathione (GSH) and thioredoxin (TRX) [42,43] (Figure 2). Mammals express six PRX isoforms, including PRX_3_ and PRX_5_ in mitochondria [41]. H_2_O_2_ oxidizes the active cysteine residue of PRXs and itself is reduced to H_2_O. The oxidized active cysteine residue can be reduced by TRX, thioredoxin reductase (TR), and NADPH for recycling; therefore, it can significantly reduce peroxide levels. GPXs exist widely *in vivo* and can catalyze the reaction between peroxide and glutathione (GSH), which protects the structure and function of cells from the interference and damage of oxides. CAT mainly exists in the peroxisome of cells, and its main role is to promote the decomposition of H_2_O_2_ into O_2_ and H_2_O. These antioxidant enzymes all play a key role in the biological defense system, but there are some differences in their functional processes. These differences come from the reaction rate (rate constant) at which they react with the substrate, as well as the concentration of substrate and enzyme [44]. In general, PRXs have a high rate constant and high abundance and therefore can eliminate nanomolar ROS related to signal transmission. GPXs have a similar rate constant to PRXs, but are less abundant, so can only act at higher H_2_O_2_ concentration, such as hypoxia, hunger and other stressful environment [44]. Thus, PRXs might be the key to turning off ROS signals, while GPXs might be the key to buffering high levels of ROS. The combined effect of the two enzymes can regulate the level of ROS precisely to ensure that cells can not only escape damage, but also initiate a signal stress response, which is crucial to maintain the homeostasis [41]. In structurally and functionally intact mitochondria, the antioxidant capacity can maintain the balance of mROS, and the reduced antioxidant capacity is one of the reasons for the increase of mROS levels and the occurrence of oxidative stress. Notably, in addition to scavenging excess ROS, the protein repair and degradation are both important defense mechanisms against ROS induced damages.

The cells also regulate the production of mROS to control their levels. However, the current understanding of this aspect is only based on experimental studies of mitochondria and cells *in vitro*, and the mechanism controlling the production of mROS *in vivo* has not yet been determined [45]. According to existing results, there are two determining factors that affect the production of mROS. One is the redox state of the ETC. Inhibition of ETC electronic carriers will increase the possibility of superoxide production. The second is the proton motive force (PMF). When the PMF increases, enhanced mROS production is observed [46]. Another study showed that the number of mitochondria also affects the production of mROS, but because of the presence of peroxisome proliferator-activated receptor gamma coactivator 1 alpha (PGC-1α), which regulates mitochondrial production and thus inhibits the production of mROS, the number of mitochondria is not necessarily proportional to the amount of mROS [47].

In addition, the signaling capacity of mROS might be altered by the mitochondria’s location. ROS are generally short-lived molecules; therefore, the coexistence of their production sites and signal functional sites may increase their efficiency.

## Physiological roles of mROS

### mROS and signal transduction

To date, many studies have shown that ROS can activate signal transduction. One of the mechanisms is ROS-mediated oxidation of amino acid residues on a target protein. These amino acid residues mainly include cysteine residues and methionine residues [48]. Phosphatase-containing active cysteine residues are one of the most studied oxidative modification sites. ROS have been proven to inhibit the activities of many phosphatases, such as angiotensin homologous enzyme and mitogen activated protein kinase. The kelch-like ECH-associated protein 1 (Keap1)/nuclear factor E2-related factor 2 (Nrf2) signaling system is one of the main signaling pathways activated by ROS via the above mechanisms. The destruction of Keap1 by ROS leads to the dissociation of the Nrf2-Keap1 complex and the activation of Nrf2.

Low doses of H_2_O_2_ can induce calcium signaling [49–51]. For example, mROS plays an important role in central nervous signal transaction. H_2_O_2_ produced by monoamine oxidase (MAO), an effective pharmacological target of the central nervous system, can stimulate lipid peroxidation, activate phospholipase C (PLC), and induce inositol 1,4,5-trisphosphate (IP3)-dependent calcium signaling. The calcium signal produced by dopamine in astrocytes is also induced by H_2_O_2_ produced by MAO [52]. It can be inferred that the activation of signal transduction by this mechanism might be a common phenomenon in nerve cells; however, further studies are needed to prove this hypothesis. ROS play an important role in the regulation of various ion channels. On the one hand, the redox environment in the cell can regulate the gated characteristics of ion channels and their activities. On the other hand, mROS can regulate the activity of amino acid residues of different channels or receptor proteins [53–55], thus mROS can control a variety of signaling pathways through the regulation of ion channels.

### mROS and aging

One of the most popular theories of aging is the ‘free radical theory’ of aging proposed by Denham Harman in the 1950s, which suggests that aging of organisms is caused by the accumulation of free radicals in cells. Free radicals, as by-products of oxidative metabolism, can cause damage to cellular proteins, lipids and DNA, resulting in the loss of the overall ability to adapt over time. Experimental phenomena such as free radical inhibitors and antioxidants can prolong the lifespan of animals or cells, and species with low free radical production have longer lifespans have confirmed this conception. However, several recent studies have shown the opposite effect, for example, several *in vivo* studies have shown that increasing the antioxidant capacity doesn’t increase lifespan. For example, it has been reported that the lifespan of C. elegans mutants lacking mitochondrial SOD was not severely affected, while SOD2 single mutants lived even longer than wild-type. After adding additional oxidative stress (caused by paraquat), SOD mutants have a shortened lifespan even much faster than killing the wild type [56]. A reasonable explanation for these interesting observations is that, in the absence of additional oxidative stress, moderate oxidative stress can induce sufficient adaptation to protect these mutants from permanent damage caused by endogenous ROS. Chen and Andziak et al. [57,58] showed that the ROS expressed by long-lived species and their accompanying oxidative damage were not always at a low level. For example, ROS prolongs the lifespan of worms [59,60]. In yeast, inhibition of target of rapamycin (TOR) increased intracellular mROS and prolonged chromosome lifespan [61]; Caloric restriction (CR) prolonged the life span of yeast by promoting the production of H_2_O_2_ [62,63]. In addition to the model organisms mentioned above, ROS has been shown to prolong the life span of human fibroblasts under hypoxia. These phenomena add more support to the above argument, but do not mean that the ‘radical theory’ is wrong, they only suggest to us that any theory claims ROS is the sole cause of biological aging is discredited. A plausible concept is to explore the protective effects of ROS on the aging of organisms [64].

Mitochondria are the main production sites of intracellular ROS, and changes in mitochondria and their functions have been regarded as the driving factors of aging. mROS can induce permanent cell cycle arrest and play a very important role in initiating and maintaining cell senescence [65,66]. However, some studies have also shown that mROS and mROS-induced mitochondrial damage can initiate signals that activate multiple pathways that protect mitochondria from stress, delay aging, and inhibit cell death. Therefore, increasing mROS levels within a certain range can prolong lifespan [67–69]. For example, Stefanatos et al. showed that an increase of mROS in the brain of mice prolonged their lifespan, indicating that mROS may also protect brain signal pathways [70]. In addition, some scholars believe that although high levels of mROS play an important role in the occurrence of neurodegenerative diseases, such as Parkinson's disease, they are not a direct cause of aging. The relationship between mROS and aging requires further extensive verification.

### mROS and stem cell differentiation

The abundance of mitochondria in stem cells is very low; therefore, these cells rely mainly on glycolysis to obtain energy. However, the importance of mROS in regulating stem cell activity and differentiation is often overlooked [71]. In recent years, research on whether mROS is required for stem cell differentiation has been carried out. Owusu-Ansah et al. found that the elimination of ROS by enhancing the expression of GTPx-1 could delay the differentiation of Drosophila multipotent hematopoietic progenitor cells. Increasing ROS by inducing the deletion of mitochondrial complex I protein ND75 or SOD2 could promote differentiation [72]. Tormos et al. showed that the differentiation of human mesenchymal stem cells (MSCs) into adipocytes could be inhibited by either knocking out the complex III protein complex III protein Rieske Iron-Sulfur Protein or using mitochondria targeted antioxidants to reduce mROS levels [73]. In addition, a study by Hamanaka et al. confirmed that mROS are important regulators of epidermal differentiation [74]. Bigarella et al. showed that mROS are also important for the differentiation of neural stem cells (NSCs) [75].

**Figure 3.**
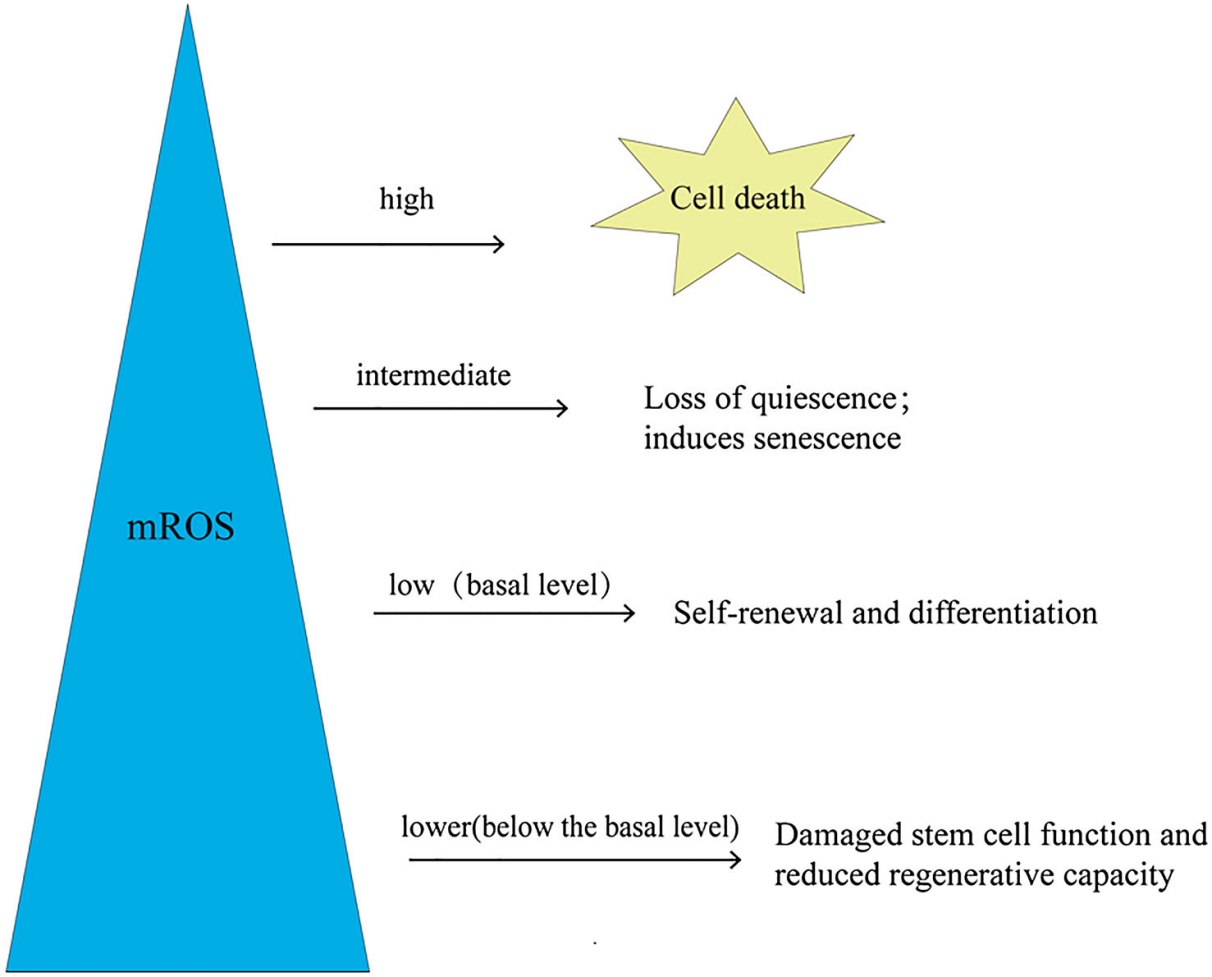
**mROS levels affect stem cell function and fate.** The level of mROS is closely related to the fate and function of stem cells. Stem cells maintain basic ROS levels to balance self-renewal and differentiation. When mROS levels are below the baseline, stem cell function is impaired and metabolic capacity is reduced. When mROS accumulate to an intermediate level, loss of immobility and the induction of senescence occur. Further accumulation of mROS to a high level leads to cell death. mROS, mitochondrial reactive oxygen species.

Mitochondrial activity significantly affects the function and differentiation of stem cells. Stem cells initially prioritize glycolysis over oxidative phosphorylation; therefore, they exhibit lower mitochondrial activity, which limits electron flux, inhibits mROS production, and maintains their regenerative potential. When metabolism is converted to oxidative phosphorylation, mitochondrial activity increases, which promotes the production of mROS and reduces stem cells’ regenerative potential [76,77]. These results indicate that the regenerative potential of stem cells is closely related to the level of mROS and the redox status *in vivo*. Lower mROS levels can maintain the balance between cell quiescent differentiation and self-renewal [78–80]. However, when mROS are lower than the basal level, a significant decrease in regeneration ability will occur (Figure 3). For example, when the levels of ROS in NSCs and hematopoietic stem cells (HSCs) were lower than the basal level, their proliferation, differentiation, and self-renewal abilities were decreased [78,81,82]. In contrast, excess ROS is associated with reduced stem cell function and regenerative potential [48,78,83–90]. For example, the long-term accumulation of ROS in HSCs severely impaired their reconstitution potential, and the stem cell pool was depleted [78,86,87]. Notably, further increases in ROS lead to cell death [91,92]. Therefore, physiological levels of mROS are necessary for stem cells to perform their normal physiological functions.

### mROS and hypoxic adaptation

When the organisms face a hypoxic environment, it will reduce oxygen consumption in various ways, and increase the supply of oxygen at the same time. Ironically, most of the adaptation pathways to hypoxia are mediated by mROS.

When cells are exposed to a low oxygen concentration for a short period of time, adaptive responses can be induced through the protein kinase pathway, which is activated by adenosine monophosphate (AMP) to promote glycolysis and increase the energy supply under hypoxic conditions. However, when the cells is subjected to chronic hypoxia, this protective measure is no longer effective, and instead, it forms an adaptation to hypoxia by stimulating HIF [93,94]. HIF is a heterodimeric transcription factor composed of an oxygen-sensitive α subunit and a constitutively expressed β subunit, and is a determinant of cell adaptation to hypoxia [95]. Under normal oxygen conditions, HIF-α is rapidly hydroxylated by prolyl hydroxylase 2 (PHD2), and the hydroxylated HIF-α subunit is degraded by the Von Hippel-Lindau tumor suppressor (VHL) [96]. In hypoxia, PHD2 is inhibited by mROS and HIF-α does not undergo hydroxylation, thus maintaining HIF homeostasis [97,98]. To date, many studies have confirmed that inhibition of PHD2 is regulated by mROS. Chandel et al. showed that the cells without mitochondrial DNA could not maintain the stability of HIF, and could not initiate a variety of transcription pathways involving HIF. Therefore, it was speculated that the stability of HIF was related to the mitochondrial electron transport chain. Subsequent studies found that the cells expressing mutant cytochrome subunits could not consume oxygen for oxidative phosphorylation, but could produce ROS and inhibit HIF degradation, which further confirmed that mROS were the main factors that stabilize HIF [99].

HIF activates the transcription of more than 70 genes *in vivo* [1]. For example, when cells are exposed to hypoxia, HIF can regulate the oxygen supply in the blood by controlling the transcription of several functional genes [100,101], such as *EPO* [102] encoding erythropoietin and *VEGF* [100] encoding vascular endothelial growth factor. HIF-1 also inhibits the conversion of pyruvate by promoting the expression of 3-phosphoinositide-dependent protein kinase-1 (PDK1) and lactate dehydrogenase A (LDHA) in cells. At the same time, acetyl-Coenzyme A promotes the conversion of pyruvate to lactic acid. The produced lactic acid can regenerate coenzyme Ⅰ, so as to carry out continuous glycolysis to provide enough energy [103–105]. HIF can also increase the concentration of glucose transporters and key enzymes in glycolysis to improve the rate of glycolysis and increase the production of anaerobic energy [103]. In addition, HIF can also alleviate the damage caused by ROS. Studies have shown that HIF protein can promote ROS clearance and enhance the oxidative defense ability by upregulating the levels of SOD2 and GSH under hypoxic conditions [106].

In addition to HIF-mediated transcriptional effects, there are other non-transcriptional effects, including increasing intracellular calcium storage, triggering the contraction of pulmonary vasculature to divert blood from hypoxic lung regions [107,108], the release of neurotransmitters by the carotid body to increase the respiration rate, and the reduction of ATP usage [41].

## Conclusion

mROS are produced in large quantities under stress conditions such as hypoxia, starvation, and pathogen infection, and are regarded as a marker for changes in the internal and external environment of the cell. The accumulation of mROS can cause damage to DNA, proteins, and lipids, and induces a variety of pathological processes. However, their effects will change with environmental alterations; therefore, antioxidants do not have a definite therapeutic effect. Physiological levels of mROS have dual functions of promoting cell damage and cell adaptation, making them a potential therapeutic target. However, due to their complex regulatory mechanisms and diverse biological functions *in vivo*, challenges remain regarding their practical.

## Authors’ contributions

CYP, CF, YJY, CQY, XXW, CJG and ZLC provide ideas and directions for article writing. BYZ read literatures and write articles. All authors read and approved the inal manuscript.
